# Evaluation of Two Methods for Determination of CD64 as a Diagnostic Marker of Infection in Critically Ill Adults

**DOI:** 10.1155/2016/6593232

**Published:** 2016-12-20

**Authors:** Thiago Zinsly Sampaio Camargo, Alexandre R. Marra, Nydia Strachman Bacal, Eduardo Casaroto, Lilian Moreira Pinto, Jacyr Pasternak, Elivane da Silva Victor, Oscar Fernando Pavão dos Santos, Michael B. Edmond

**Affiliations:** ^1^Intensive Care Unit, Hospital Israelita Albert Einstein, São Paulo, SP, Brazil; ^2^Instituto Israelita de Ensino e Pesquisa Albert Einstein, Hospital Israelita Albert Einstein, São Paulo, SP, Brazil; ^3^Clinical Laboratory, Hospital Israelita Albert Einstein, São Paulo, SP, Brazil; ^4^Division of Medical Practice, Hospital Israelita Albert Einstein, São Paulo, SP, Brazil; ^5^Department of Internal Medicine, University of Iowa Carver College of Medicine, Iowa City, IA, USA

## Abstract

*Objectives*. Diagnostic markers of infection have had little innovation over the last few decades. CD64, a marker expressed on the surface of neutrophils, may have utility for this purpose.* Methods*. This study was conducted in an adult intensive care unit (ICU) in São Paulo, Brazil, with 89 patients. We evaluated CD64 in patients with documented or clinically diagnosed infection (infection group) and controls (patients without any evidence of infection) by two different methodologies: method #1, an in house assay, and method #2, the commercial kit Leuko64 (Trillium Diagnostics).* Results*. CD64 displayed good discriminating power with a 91.2% sensitivity (95% CI 90.7–91.6%) for detecting infection. The commercial kit (Leuko64) demonstrated higher specificity (87.3%) compared with method #1 as well as better accuracy (88.8%).* Conclusions*. CD64 seems to be a promising marker of infection in the intensive care setting, with Leuko64 showing a slight advantage.

## 1. Background

Sepsis is a worldwide public health problem, with an estimated 750,000 new cases of severe sepsis and septic shock diagnosed each year in the US, with associated short-term mortality about 20% or more, making it one of the top ten causes of death in the intensive care unit (ICU) [1–3].

Diagnostic markers of systemic inflammatory response and sepsis have shown few innovations over the last few decades, despite advances in molecular science and the cell biology of myeloid effector cells and cytokines involved in the innate immune response [4].

Laboratory tests available to diagnose infection and sepsis are those dating from the 1970s or older, such as neutrophil counts, identification of immature myeloid cells in the peripheral blood, and acute phase reactants like C-reactive protein (CRP). Currently, we have seen efforts to develop a novel biomarker, procalcitonin, that may improve the diagnosis of infection and sepsis [5].

Some studies have shown that the quantitative expression of CD64 (high affinity Fc receptor) in polymorphonuclear neutrophils (PMN) could be used as a more sensitive and specific marker to confirm or exclude sepsis [6–8]. It has also been validated in some studies as a specific biomarker for bacterial infections in the ICU, showing good discriminatory power to differentiate sepsis of bacterial, viral, or fungal origin from other inflammatory conditions [9].

CD64 is the high affinity receptor for IgG and is involved in antibody-dependent cell-mediated cytotoxicity, phagocytosis, and regulation of cytokine production. Monocytes and macrophages also express CD64, while mature granulocytes and lymphocytes are negative.

In the ICU, it could be used at admission in diagnosis infection, or for monitoring purposes with serial determinations [10].

Outside the ICU, its use has been attempted to differentiate infection versus noninfectious inflammation in localized sites, such as septic arthritis versus noninfectious joint inflammation, but except in patients with concurrent bacteremia, its sensitivity and specificity have been less than optimal [11].

The primary objective of this study was to assess the clinical use of CD64 as a diagnostic marker of infection in ICU patients. The secondary objective was to define the cutoff value to discriminate between the studied groups by the different methods.

## 2. Methods

This prospective cohort study included patients admitted to the ICU of a tertiary hospital in São Paulo, Brazil. This study was approved by the Institutional Review Board (IRB) of Hospital Israelita Albert Einstein. Patients eligible to participate in the study were those admitted at the ICU every Tuesday and Thursday during the study period (April 2010 through May 2011) and who gave written informed consent. If the patient could not provide written informed consent, the legally responsible family member for the admission did it. Patients with end-stage cancer, solid organ transplant, and HIV infection or those actively dying were excluded. Within 60 minutes after their admission at the ICU, a blood sample was obtained for laboratory tests, including CD64 determination.

At the end of the hospitalization period, a blinded investigator assessed the patients' records to categorize patients into two groups: (1) septic patients with a microbiologically documented infection or with clinical/radiographic evidence of infection according to two different examiners; and (2) control patients, that is, those without any evidence of infection.

The diagnosis of infection followed the International Sepsis Forum [12] guidelines. Patients who had undergone surgery within 4 weeks of admission were considered surgical cases. Elective surgery was defined as that scheduled at least 24 hours in advance. Trauma related admissions were those directly related to the event or occurring as a complication of a traumatic event within the past 30 days. All other admissions were considered medical [13].

The following data were recorded for patients in both groups: age, gender, clinical status at ICU admission (septic patients with documented infection or control patients without infection, at the end of hospitalization a blind investigator); clinical predisposing conditions, underlying diseases such as neoplasm, renal failure, and diabetes mellitus; ICU length of stay (days); antimicrobial therapy (yes, no, or antimicrobial prophylaxis); and hospital mortality. The clinical status of each patient was assessed according to the systemic inflammatory response syndrome criteria (SIRS, sepsis, severe sepsis, or septic shock) and according to the severity scores, SOFA [13] and APACHE II [14], at admission. The Charlson criterion was also included [15].

The study investigators had no interference in the clinical conduction of the patients; at the same time, assistant physicians did not have knowledge of CD64 for making their clinical decisions.

All data were collected on a standardized form without any patient identifying information and were kept strictly confidential. Patients were followed up until hospital discharge and assessed based on their medical records while hospitalized. Patients, their physicians, and/or their legal representatives had access to all information as required.

### 2.1. CD64

CD64 measurement was performed on the same blood sample collected routinely for CBC at admission (1.0 mL), in most cases without the need for additional venipuncture. In some instances, a blood sample was obtained specifically for CD64 determination.

For method #1, phosphate-buffered saline-diluted whole blood (50 *μ*L) was incubated for 15 min at room temperature with a combination of CD64 FITC- (fluorescein isothiocyanate-) clone IM1604u (Beckman Coulter) and CD45 PE- (phycoerythrin-) clone IM2078 (Beckman Coulter). After lysis of red blood cells, samples were washed and fixed.

CD64 was assessed in neutrophils separated by marking with CD45, using the CD45x Side Scatter graphic (SSC). The result was assessed by calculating the ratio between the Mean Gene Expression (MnX) obtained from the patient and the MnX obtained from the control. The result expressed in log scale was directly related to the antigenic density of the CD64 monoclonal antibody on the cell surface.

The readings were done on Cytomics FC500 (Beckman Coulter).

For method #2, the commercial kit Leuko64 (Trillium Diagnostics, LLC) was used and readings were done on the Cytomics FC500 (Beckman Coulter) equipment. The results were assessed with the QuantiCALC software. According to the instructions in the kit, the expected index in normal subjects is PMN CD64 ≤ 1.00.

### 2.2. Statistical Analysis

To assess the correlation between markers and infection, logistic regression models were fit. The occurrence of infection was the dependent variable and the markers individually were evaluated as independent variables; ROC curves were also constructed. The models' goodness of fit assessment was done using the Hosmer and Lemeshow test and graphs for leverage, Cook's distance, deviation component, and residual deviation component. The cutoff point for the measurements was defined as the point with the highest specificity among those with at least 90% sensitivity. Once the cutoff points defined the utility of CD64 read by the two different methodologies, measurements of accuracy, sensitivity, and specificity were assessed, as well as the kappa coefficient for agreement.

All measurements were shown with their 95% confidence intervals. For the comparison of numerical measures in the groups, the Mann–Whitney* U* test was used.

The analyses were performed using the R applications (R Core Team (2012), https://www.r-project.org/, version 2.15.1) and SPSS (SPSS Inc. Released 2008. SPSS Statistics for Windows, version 17.0, Chicago: SPSS Inc.) and the significance level was 5%.

## 3. Results

Samples from 89 patients admitted to the adult ICU during the study period were analyzed: 62.9% (*n* = 56) were males and 37.1% (*n* = 33) were females. The mean age was 65 years (SD ± 19 years). Of the total, 61.8% (*n* = 55) were classified as control patients and 38.2% (*n* = 34) were patients with infection.  Table 1 shows the patients' demographic data. In the infection group, 61.8% of the cases (*n* = 21) were microbiologically documented, and the infectious agents are shown in Table 2. The agents were isolated from the following sites: 8 from respiratory tract, 7 from urine, 4 from blood cultures, and 2 from abscesses.

At the time of sample collection, 44.9% (*n* = 40) were under treatment with some form of antimicrobial regimen for a potential infection; 24.7% (*n* = 22) were receiving antimicrobial prophylaxis (postsurgical procedures); and 30.3% (*n* = 27) were not receiving antimicrobials. In-hospital mortality was 13.5% (*n* = 12) and the diagnoses were grouped as shown in Table 3.

Logistic regression models show that the probability of infection increases significantly with each unit increase in CD64 by method #1 or method #2 (*p* < 0.001). For readings obtained with the first one, the odds ratio was estimated at 2.76 (95% CI 1.72–4.43, *p* < 0.001) and for the Leuko64 kit, the odds ratio was estimated at 6.67 (95% CI 2.79–15.96, *p* < 0.001) (Table 7).

**Figure 1:**
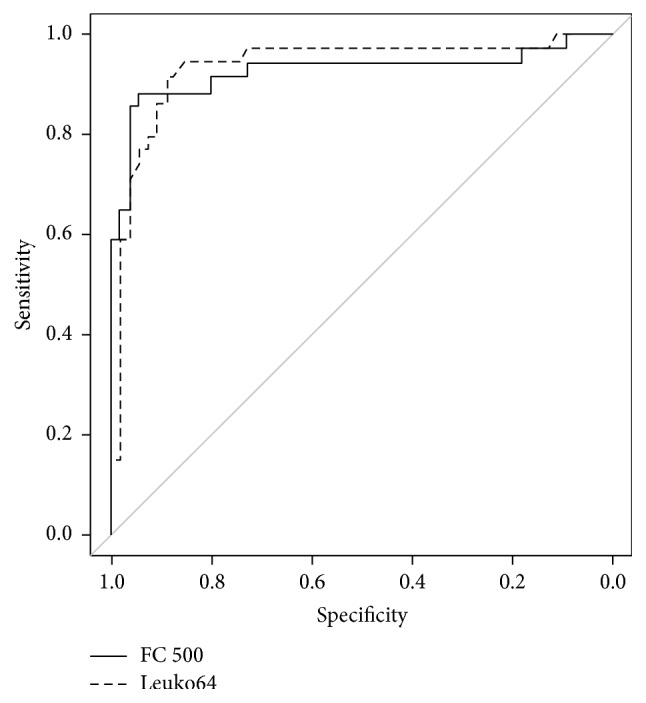
ROC curve comparing method #1 with method #2 (Leuko64) in the determination of infection markers regardless of microbiologic confirmation.

**Figure 2:**
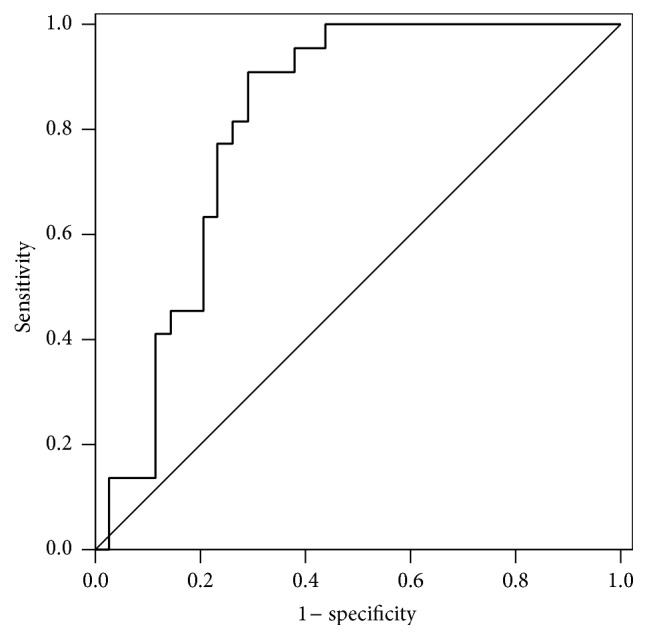
ROC curve for Leuko64 in the subgroup with documented infection; the only method studied where the AUC was significantly greater than 0.5 (*p* < 0.001).

After checking for a significant correlation between the CD64 read by the two methods, measurements, and the presence of infection using logistic regression modeling, we constructed ROC curves (Figures 1 and 2), seeking to identify cutoff points for the two markers and compare their performances as predictors of infection.

For all two markers, the area under the curve (AUC) was significantly greater than 0.5 (*p* < 0.001). The AUC obtained with method #1 was 0.925 (CI 0.853–0.997); with method #2, the AUC was 0.933 (CI 0.872–0.995). While the Leuko64 shows the highest AUC, we cannot conclude that it performs better as a predictor of infection than either of the instruments used in method #1, because the curves are close and the confidence intervals overlap each other.

Additionally, the results obtained by CD64 read were categorized based on the cutoff that maximized their specificity, ensuring at least 90% sensitivity, in fact, 91.2% (95% CI 90.7–91.6%) in this study, as show in Table 4. Based on the categorized markers, specificity, kappa agreement coefficients, and measures of performance in predicting infection were calculated. In a separate analysis, we considered only infections with microbiological confirmation as outcomes compared to control patients. Those patients with clinical symptoms of infection but without positive cultures were excluded from analysis. Logistic regression models were fit with sepsis as the dependent variable and the markers individually as independent variables. The results show that the probability of sepsis increases significantly with each unit increase in the Leuko64 reading (*p* = 0.037). For readings obtained with method #1, the odds ratio was estimated at 1.05 (95% CI 0.99–1.11, *p* = 0.1305) and for method #2, the odds ratio was estimated at 1.35 (95% CI 1.02–1.80, *p* = 0.037) (Table 7). For none of the marker measurements did the Hosmer and Lemeshow statistic show good quality of fit (method #1: *p* = 0.030, and method #2: *p* = 0.024).

After checking for a significant correlation between the Leuko64 measurement and documented sepsis using logistic regression modeling, we constructed an ROC curve (Figure 2), seeking to identify the cutoff point for the marker and assess its performance as a predictor of infection.

For the Leuko64, the area under the curve (AUC) was significantly greater than 0.5 (AUC 0.811, CI 0.698–0.925, *p* < 0.001). Additionally, the marker was categorized based on the cutoff that maximized its specificity, ensuring at least 90% sensitivity. Based on the categorized marker, the kappa agreement coefficient and measures of performance in predicting documented sepsis were calculated, as shown in Table 5.

**Figure 3:**
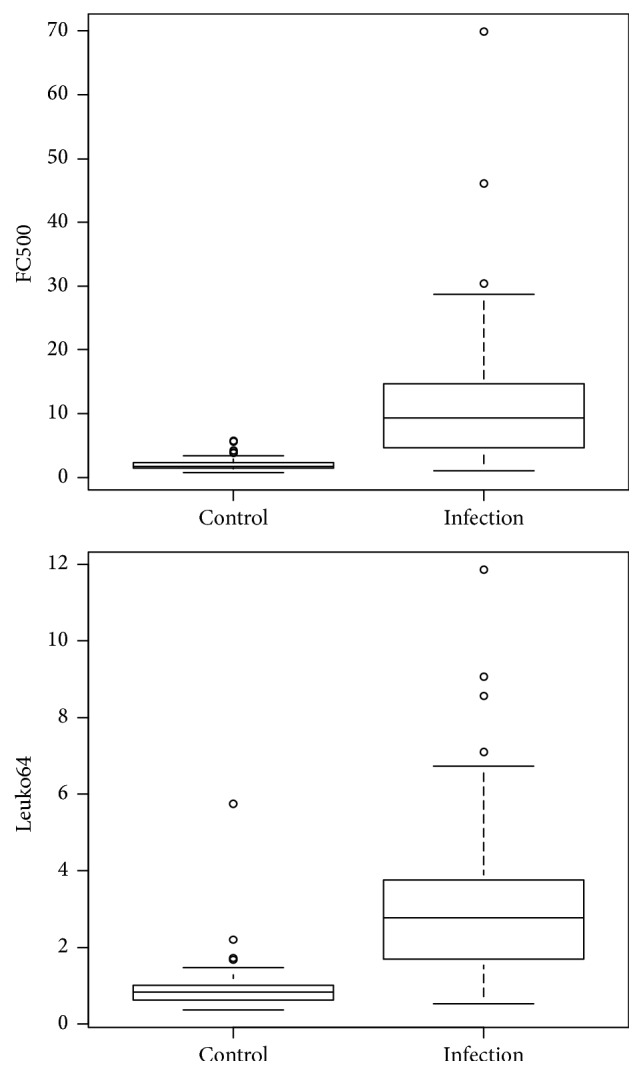
Boxplot distribution of the CD64 index according to the methods studied (FC500 and Leuko64).

Results of CD64 index can be seen in Table 6 and the boxplot distribution in Figure 3.

## 4. Discussion

Early initiation of antibiotics has been proven to be a key strategy in decreasing infection related mortality rates. However, the lack of new antimicrobial drugs to treat the increasingly resistant bacteria present in hospitals underscores the need for a diagnostic test with adequate sensitivity and specificity to guide therapeutic decisions.

Based on the defined cutoff points, CD64 has shown good discriminating power for patients with infection, with a 91.2% sensitivity (95% CI 90.7–91.6%). A recent meta-analysis of 14 studies conducted in different populations (neonates, children, and adults) and different clinical settings showed 79% sensitivity (CI 95% 70–86%), 91% specificity (CI 95% 85–95%), and AUC 0.94. In our study, the specificity ranged from 78.2% (method #1) to 87.3% (method #2). In the abovementioned meta-analysis, the specificity of CD64 was 92% (95% CI 90–95%) with an AUC of 0.95 using the in-house methodology and 75% (95% CI 52–89%) with an AUC of 0.73 using commercial kits [5]. The better performance observed in our study was perhaps due to the fact that only adults were included; in the meta-analysis by Cid et al. [5], sensitivity, specificity, and AUC were lower when the biomarker was evaluated in children and neonates as compared to adults. If we extract only the adult sample (726 patients) from this paper, we find sensitivity data that are very similar to ours and higher specificity values: 90% sensitivity (95% CI 0.75–0.96), 95% specificity (95% CI 0.92–0.97), and an AUC of 0.97.

Previous studies conducted in the ICU setting using the Leuko64 method have shown lower sensitivity (approximately 63%), with the index adjusted to >2.2 for bacterial infections [9]. Of note, in the present study, we adjusted the Leuko64 index to >1.3 for higher power of discrimination between groups. When we analyzed only patients with documented infection, the Leuko64 method yielded an AUC significantly greater than 0.5, which was not seen by the other methods used in this subgroup. Therefore, the cutoff for this method went from 1.3 in the infection group (documented or not) to 1.45 in the documented-only infection group. Consequently, higher Leuko64 values were associated with a better chance of isolating an infectious agent in the studied conditions.

Several confounding factors may explain these differences between studies; they relate to not only population and methodologies but also how biomarkers are handled in the lab. For example, the timing of sample collection is important as some authors have shown that CD64 upregulation for expression on the neutrophil surface occurs as quickly as 4 to 6 hours. In addition, the meta-analysis pointed out the low quality of the methodology used in some of the studies included.

False-negative and false-positive results may be related to the different cutoff values of the reported CD64 tests. In some studies, a wide variation in sensitivity and specificity was reported, with a wide range of cutoff values even if the same method for the measurement of CD64 expression had been employed. Increases in CD64 are demonstrable within 4–6 h of neutrophil contact with proinflammatory cytokines, with peak expression observed >48 h. Normalization of increased neutrophil CD64 expression during antibiotic therapy has been reported, indicating the importance of measuring neutrophil CD64 expression at the correct time.

In our study, the index detection with the commercial kit (Leuko64) showed higher specificity (87.3%) compared with the other method, as well as better accuracy (88.8%). Of note, the negative predictive value of the test with any of the methods tested was >93%, which demonstrates the potential utility of using the test in the decision of whether to initiate antibiotic therapy.

Another more recent meta-analysis also assessed CD64 as a biomarker for early diagnosis of infection and concluded that this is a promising tool. However, the different cutoffs used across the studies generate controversy concerning its clinical use. This meta-analysis included 26 studies with a total of 3,944 patients, also with a mixed population of neonates, children, and adults, and estimated the test sensitivity at 76% (CI 95% 0.74–0.78) and its specificity at 85% (CI 95% 0.83–0.86). Again, the studies included showed great heterogeneity [16].

Our study has some limitations, including the relatively small sample size, no comparison with C-reactive protein (CRP), procalcitonin levels, and the performance in a single medical center, as well as the exclusion of solid organ transplant patients that are increasingly present in ICUs. The unsatisfactory result of the Hosmer-Lemeshow test for infections with microbiological confirmation is due to the fact that we have not included in the model other potentially influential factors to the outcomes. The lack of fitting to the models was not addressed in this study since it does not aim to predict the occurrence of outcomes based on the results by adjusted logistic regression models but seeks to identify relationships between markers and events for later construction of the ROC curve and detection of cutoffs, which were evaluated by sensitivity, specificity, and other metrics. On the other hand, the analysis considering the markers categorized by cutoffs showed that the markers were accurate in the identification of outcomes.

For the direct determination of the CD64 monoclonal antibody, method #1, there was no need to change the gains and voltages of the photomultipliers (fluorescence uptake and reading sites); therefore, the test could be performed in a daily routine setting. With the Leuko64 kit (method #2), it is necessary to change the flow cytometer calibration using the beads supplied with the kit, thereby defining new gains and voltages for subsequent analysis in the Leuko64 application; such calibration must be repeated for each new kit batch. Besides the calibration changes, the Leuko64 kit requires specific training of the technical staff performing the test.

Based on cost of reagents, using method #1 equipment costs approximately 60% less than the Leuko64 kit (method #2). Also, the Leuko64 kit is an imported product, which represents an additional inconvenience.

This study shows that CD 64 seems to be a promising marker of infection in the intensive care setting. It may be tested by one of two methodologies, with Leuko64 showing a slight advantage. Particularly in the subgroup of microbiologically documented infections, the performance of Leuko64 was superior. Further studies should be conducted to assess this marker and compare its performance to other markers currently available in clinical practice, such as CRP [11] and procalcitonin.

## Figures and Tables

**Table 1:** Demographic data from 89 patients admitted at the ICU during the study period from which CD64 samples were collected.

	*N*	%
Gender		
Male	56	62.9%
Female	33	37.1%
Mean age (SD)	65	19
Classification		
Control	55	61.8%
Infection	34	38.2%
Microbiologically documented infection		
No	13/34	38.2%
Yes	21/34	61.8%
Antibiotic use		
Yes	40	44.9%
No	27	30.3%
Prophylaxis	22	24.7%
Charlson		
0	23	25.8%
1	24	27.0%
2	20	22.5%
3	15	16.9%
4	5	5.6%
5	2	2.2%
Death		
No	77	86.5%
Yes	12	13.5%

**Table 2:** Bacteria isolated from patients with microbiologically documented infection in the study (*n* = 21).

Bacteria	Number of cases	Site (*n*)
*Burkholderia cepacia*	1	Lung (1)
*Candida krusei*	1	Blood (1)
*Candida tropicalis*	1	Urine (1)
*Enterococcus faecalis*	2	Lung (1), urine (1)
*Escherichia coli*	7	Blood (2), urine (5)
*Klebsiella pneumoniae*	2	Lung (1), abscess (1)
*Peptoniphilus asaccharolyticus*	1	Abscess (1)
*Pseudomonas aeruginosa*	1	Lung (1)
*Serratia marcescens*	2	Lung (2)
*Staphylococcus aureus*	3	Lung (2), blood (1)

**Table 3:** Groups of diagnoses in the studied population (*n* = 89).

Diagnosis	*N*	%
Sepsis/septic shock	18	20.2%
Other shock states	1	1.1%
Cardiovascular disease	10	11.2%
Respiratory failure	11	12.4%
Acute renal failure	1	1.1%
Neurologic disease	16	18.0%
Postoperative (neuro/cardio)	6	6.7%
Transplant (bone marrow)	1	1.1%
Multiple trauma	1	1.1%
Other	24	27.0%

**Table 4:** Utility of CD 64 markers categorized according to the cutoff that for each method maximized specificity ensuring at least 90% sensitivity [actually 91.2% (95% CI 90.7–91.6%)].

	Method #1	Method #2
AUC	0.925 (0.853–0.997)	0.933 (0.872–0.995)
Cutoff	2.4	1.3
After categorization		
TP	31	31
FP	12	7
TN	43	48
FN	3	3
Specificity	78.2% (77.6%–78.8%)	87.3% (86.9%–87.7%)
Kappa	0.66 (0.55–0.77)	0.77 (0.67–0.86)
Accuracy	83.2% (82.8%–3.5%)	88.8% (88.5%–89.0%)
PPV	72.1% (71.2%–73.0%)	81.6% (80.8%–82.4%)
NPV	93.5% (93.2%–93.7%)	94.1% (93.9%–94.3%)

AUC: area under the curve; TP: true positive; FP: false positive; TN: true negative; FN: false negative; PPV: positive predictive value; NPV: negative predictive value.

**Table 5:** Characteristics of the Leuko64 test after checking for significant correlation with documented infection using logistic regression modeling.

	Leuko64
AUC	0.811 (0.698–0.925)
Cutoff	1.45
After categorization	
TP	20
FP	10
TN	24
FN	2
Kappa	0.58 (0.43–0.73)
Accuracy	78.6% (78.0%–79.2%)
PPV	66.7% (65.2%–68.1%)
NPV	92.3% (91.8%–92.8%)

AUC: area under the curve; TP: true positive; FP: false positive; TN: true negative; FN: false negative; PPV: positive predictive value; NPV: negative predictive value.

**Table 6:** CD64 index according to methodology and classification of patients.

		Classification		Mann–Whitney *U* (*p*)
		Control	Infection	
FC500	Minimum	0.78	1.02	<0.001
	Median	1.76	9.25	
	Maximum	5,77	69.90	

Leuko64	Minimum	0.37	0,52	<0.001
	Median	0.82	2.78	
	Maximum	5.74	11.86	

**Table 7:** Logistic regression for the probability of infection with each unit increase by the different methods studied.

	Odds ratio	CI 95%	*p*
Infection group			
FC500	2.76	1.72 a 4.43	<0.001
Leuko64	6.67	2.79 a 15.96	<0.001
Subgroup of documented infection			
FC500	1.05	0.99 a 1.11	0.131
Leuko64	1.35	1.02 a 1.80	0.037
